# Social communication activates the circadian gene *Tctimeless* in *Tribolium castaneum*

**DOI:** 10.1038/s41598-021-95588-1

**Published:** 2021-08-09

**Authors:** Animesha Rath, Miriam Benita, Josef Doron, Inon Scharf, Daphna Gottlieb

**Affiliations:** 1 Department of Food Science, Institute of Post-Harvest and Food Science, The Volcani Center, ARO, Rishon LeZion, Israel; 2 School of Zoology, Faculty of Life Sciences, Tel Aviv University, Tel Aviv, Israel

**Keywords:** Chemical biology, Ecology, Molecular biology, Zoology

## Abstract

Chemical communication via pheromones is an integral component in insect behavior, particularly for mate searching and reproduction. Aggregation pheromones, that attract conspecifics of both sexes, are particularly common and have been identified for hundreds of species. These pheromones are among the most ecologically selective pest suppression agents. In this study, we identified an activating effect of the aggregation pheromone of the red flour beetle, *Tribolium castaneum* (Herbst) (Coleoptera: Tenibroidae) on a highly conserved circadian clock gene (*Tctimeless*). *Tribolium castaneum* is one of the most damaging cosmopolitan pest of flour and other stored food products. Its male produced aggregation pheromone, 4,8-dimethyldecanal (DMD), attracts both conspecific males and females and is used for pest management via monitoring and mating disruption. The *Tctimeless* gene is an essential component for daily expression patterns of the circadian clock and plays vital roles in eclosion, egg production, and embryonic development. In this study, we demonstrate that constant exposure to the species-specific aggregation pheromone led to *Tctimeless* up-regulation and a different pattern of rhythmic locomotive behavior. We propose that changing the well-adapted "alarm clock", using DMD is liable to reduce fitness and can be highly useful for pest management.

## Introduction

The red flour beetle, *Tribolium castaneum* (Herbst) (Coleoptera: Tenebrionidae), larvae and adults are attracted to damaged grains or other farinaceous materials. These beetles are highly adapted to the stored-grain environment, with high fecundity and relative longevity^1^. Their well-developed ability to move among multiple food patches contributes to their rapid infestation of newly established resources, such as stored grains^2^. The main attractants of the beetle to the grain storage are volatile stimuli that originate from the stored food^3^ and the conspecific aggregation pheromones^4^. *Tribolium castaneum* males attract both sexes via the aggregation pheromone, 4,8-dimethyldecanal (DMD)^5–8^. Chemical control is one of the most efficient methods to control this pest and prevent economic loss^9^. However, the frequent use of pesticides has led to the emergence of resistant strains^10,11^. This situation has led to the search for alternative methods to control the pest, such as the use of pheromones, since pheromones mediate the major channel of communication in insect mate-finding^12,13^. Based on the knowledge gained on DMD, several integrated pest management strategies have been developed for *T. castaneum*. Amongst these strategies, the most common use of DMD is in pheromone-baited traps^14^. However, these traps have achieved only a limited success^15^ and were inconsistent in their sex differential attraction, with females responding more strongly^16,17^, equally^18,19^, or less strongly^20,21^ than males.

We offer here a new aspect to the questionable success of these traps, by suggesting that the DMD affects the circadian clock, and thus, reduces the species fitness. In insects, as in most organisms, the circadian clock system is a major regulatory factor for nearly all physiological and behavioral activities^22^. There is also accumulating evidence that social interactions in insects alter the circadian clock rhythmicity^23^. Thus, studying the effect of DMD, which is a social cue, on the circadian clock may reveal the broad effect of DMD on the life-history traits of the beetles. There is currently only little information on the circadian clock of *T. castaneum*^24,25^. The main "clock" genes identified are *Tctimeless* (*Tctim*), *Tcperiod* (*Tcper*), *Tcclock* (*Tcclk*), *Tccryptochrome-2* (*Tccry2*), and *Tccycle* (*Tccyc*). Positive elements of CYC and CLK (product proteins of *Tccyc* and *TcClk*) form a heterodimer and subsequently activate the transcription of clock genes *per* and possibly many other genes called clock-controlled genes (*ccg*). Their protein products are synthesized and PER, TIM and CRY2 (product proteins of *Tcper*, *Tctim*, *Tccry2*) form a complex. The complex acts as a negative element that represses the CYC/CLK transcriptional activity. Reduction of *ccg* transcript levels and consequent reduction of CCG protein levels lead to a decrease of repressive regulation of CYC/CLK by PER/TIM/CRY2, and therefore CYC/CLK-mediated transcription increases again. These phases, in which *ccg* transcription is activated or repressed, are repeated in an approximately 24 h cycle. CRY2 (also called mammalian-type *cry*) does not function as a photoreceptor (as *cry1* *D. melanogaster*), but contributes to the circadian feedback loop as a negative element by forming a complex with PER and TIM to suppress the CLK/CYC activity. Light input may nevertheless be mediated through the degradation of *Tctim*^25,26^.

There are several circadian clock outcomes that have been studied, including the DMD daily periodic release^20,21^. Here we study the potential feedback effect of the pheromone to the molecular circadian clock. We initially evaluated the peak hours of circadian gene expression (*Tctim* and *Tcper*). Then, we evaluated the direct effect of the synthetic pheromone on *Tctim* expression at the peak hours found in the initial experiment. We focused on *Tctim* since it plays a vital role in the successful completion of eclosion, egg production, and embryonic development^24^. Thus, any effect of DMD on *Tctim* may involve various aspects of life-history traits and fitness of *T. castaneum*. The molecular study was followed by an evaluation of the effect of the synthetic pheromone on the rhythmicity of locomotive behavior.

## Materials and methods

### Insects rearing

*Tribolium castaneum* beetles were collected from commercial grain storages in the northern galilee region, Northern part of Israel*.* The beetles were reared on broken wheat grains and were entrained to a regime of a 12:12 h light–dark (L:D) for two weeks at 25 °C and 65 ± 5% relative humidity.

### Oscillation of Tctimeless and Tcperiod

We studied the oscillation of *T. castaneum* clock genes (*Tctim* and *Tcper)* in groups of four and eight individuals (1:1 sex ratio). To get optimum and good quality nucleic acid, RNA was extracted from a pool of four beetles' heads (n). In total there were three biological replicates (n = 3) for each time point for each group (four and eight). After cDNA synthesis, the samples were subjected to quantitative real time-PCR to study the daily expression of the clock genes (described in Methods and Materials: Gene expression analysis).

### Exposure to DMD

In the molecular and behavioral experiments, the exposure of beetles to synthetic DMD was achieved by exposing them to Rubber septa, pheromone commercial lures with a surplus amount of synthetic DMD (Pherobees: Tribolur). To imitate the practiced use of the pheromone traps, the beetles were exposed continuously to DMD throughout the experiment. As in most pheromone scented traps, we assumed that the synthetic DMD (Pherobees: Tribolur)^20,21^ has higher concentration (approximately × 10^6^) throughout the day than the daily peak of the naturally emitted DMD (878 ng per male daily^28^). The experimental design is described below separately for the molecular and behavioral experiment.

#### Molecular experiment

**Figure 1:**
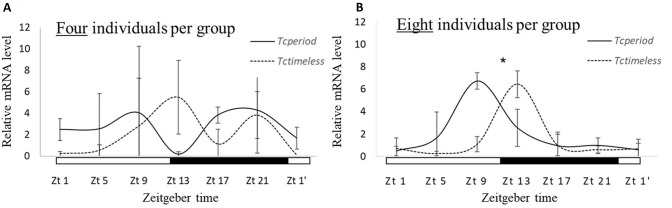
*Tctim* and *Tcper* expression (mean ± SD) in groups of four (**A**) and eight (**B**) individuals. The expression of *Tctim* and *Tcper* peaks only in individuals originating from the group of eight (**B**). White and black bars indicate light phase and night, respectively. *indicates the peak of gene expression with a significant rhythmicity.

Prior to the experiment, beetles collected from the initial rearing glass bottles (see insect rearing) were kept in random group sizes (Range of 25–100 individuals of mixed sex, in total 20 groups). The groups were habituated, for two days in a chemical hood system, to 12:12 h light–dark regime at room temperature of 25 °C. After habituation, two days before RNA extraction, the groups were either exposed to the synthetic pheromone (hereafter DMD treatment group) or served as a control with no exposure to DMD (hereafter control group). Based on the initial experiment estimating oscillation characteristics of *Tctim*, sample collection was predetermined to 19:00 (Zt13, see Results and Fig. 1). The samples were collected during two successive days. In both treatment and control, four beetle heads of the same sex were pooled per sample (n). The eventual sample size of the pheromone exposed group was n = 6 and 3 and the control sample size was n = 11 and n = 14 for males and females, respectively.

### Gene expression analysis

Total RNA extraction and cDNA synthesis- Total RNAs were isolated from samples following a modified manual extraction protocol using RiboEx™ solution (GeneAll, Germany). The final elution was made at a volume of 40 μl using DEPC-treated water. The elution was made twice at a volume of 20 μl to ensure maximum recovery. Immediately following the RNA extraction, the samples were treated for gDNase and subsequently subjected to synthesis of first strand cDNAs from 1 μg of total RNA. gDNase treatment and cDNA synthesis was done using FastQuantRT Kit (TIANGEN, Cat no. KR106).

Primer design- Based on the genome assembly Tcas5.2 (whole genome sequence of *Tribolium castaneum*/ Ensemble Metazoa), we designed a set of primers to quantify the expression of the clock gene, *Tctim* (Table 1). Primers were designed considering a length of 20–22 base pairs and GC content of 45–55%. The SnapGene primer designing tool was used to evaluate the Tm and possibility of secondary structure formation of the designed primers. To make sure the primers were singletons, primer sequences were blast in ensemble genome of *Tribolium castaneum* for single hit. Primers were designed between consecutive exonic regions (exon 1 –exon 2, Table 1). Housekeeping gene primers for ribosomal protein L3 (*rps3*) were used from the literature described by^24^.

**Table 1:** List of primers used.

Gene	Primer name	Sequence (5’-3’)	Fragment size (bp)	Nature
*Tctimeless*	TIM F1	CTGTGTCCGAAATGGGGACA	124	cDNA
	TIM R1	GAAACCGATGGCTCTTCTGT		
*Tcperiod*	PER F1	ACAGTGGGAGTAACTCGAGT	91	cDNA
	PER R1	CGTTTAGGTGGTTGGAATCC		

Quantitative real time-PCR- To quantify the expression of circadian genes, quantitative real time-PCR (qPCR) was performed with 2X qPCRBIOSyGreen Blue MixHi-ROX (PCR Biosystems, UK). For normalizing the copy number of the studied clock gene, *T. castaneum* ribosomal protein S3 (*rps3*) was used as a reference gene for qPCR analysis. The mRNA levels were expressed in terms of relative expression using the delta CT (Cross Threshold, the PCR cycle number that crosses the signal threshold) method^28^. The CT of the *rps3* gene was subtracted from the CT of the target gene to obtain ∆CT. The normalized fold changes of the target gene mRNA expression were expressed as 2^−∆∆^CT, where ∆∆CT is equal to ∆CT_treated sample_ – ∆CT_control_. Three biological replication samples were used to measure the expressed gene levels by qPCR. Each biological replicate had three repeats for the accuracy of the results.

#### Behavioral experiment

768 T*. castaneum* beetles were kept singly in eight 96 well plates. After 24 h of acclimation under 12D:12L, we added the synthetic DMD pheromone (see details in Exposure to DMD) to half of the beetles (i.e. treatment). To avoid any handling effect of the beetles while adding the DMD, we handled the control group similarly, but without adding the DMD. After 24 h of acclimation, we started to film for 5 days. The locomotive activity (henceforth activity) was analyzed every 4 h for 2 min with AnTracks® tacking program (https://sites.google.com/view/antracks). We then compared the activity level (length of travel path/2 min) and rhythmicity (circadian activity rhythm) between the treatment and the control.

### Statistical analysis

We compared the effect of exposure to synthetic aggregation pheromone on *Tctim* gene expression levels of males and females using a generalized linearized model (GLM). Group size, sex, and treatment were treated as explanatory factors and the transcript number as the response variable. Statistics were performed using the R Core Team (2017). We used ‘lme4’^29^, ‘lmerTest’^30^ and ‘MuMIn’^31^ packages for GLM.

Gene expression values were analyzed for rhythmic oscillations using the JTK_CYCLE test^32^. A profile is considered as circadian within the interval of 24–28 h. Genes were considered to display rhythmicity at a significance threshold of BH.Q < 0.05. The Q-value was estimated by the Benjamini–Hochberg procedure.

We compared the effect of exposure to synthetic aggregation pheromone on activity levels using a Non-Normal Repeated Measurements Models, with repeated days, as the within-subject variable, treatment (DMD and control) as the between-subject variable, and activity level as the response variable. To estimate the general effect of scotophase vs. photophase, we pooled activity data from Zt1 to ZT 9, i.e. photophase and Zt 13 to Zt 21, i.e. scotophase. Statistics were performed using the R Core Team (2017). We used 'repeated'^33^ package for the repeated measurement model.

### Ethics approval

This article does not contain any studies with human participants or animals performed by any of the authors.

## Results

### Oscillation of tctimeless and tcperiod

Gene expression of both *Tctim* and *Tcper* in individuals originating from groups of four did not demonstrate significant rhythmicity (*P* > 0.5, Fig. 1A). In individuals originating from a group of eight, JTK cycle analysis revealed a significant rhythm of *Tctim* (*P* = 0.023) and *Tcper* (*P* = 0.027) with a peak of gene expression at Zt 13 and Zt 9, respectively (Fig. 1B).

### Exposure to DMD

#### Molecular experiment

**Figure 2:**
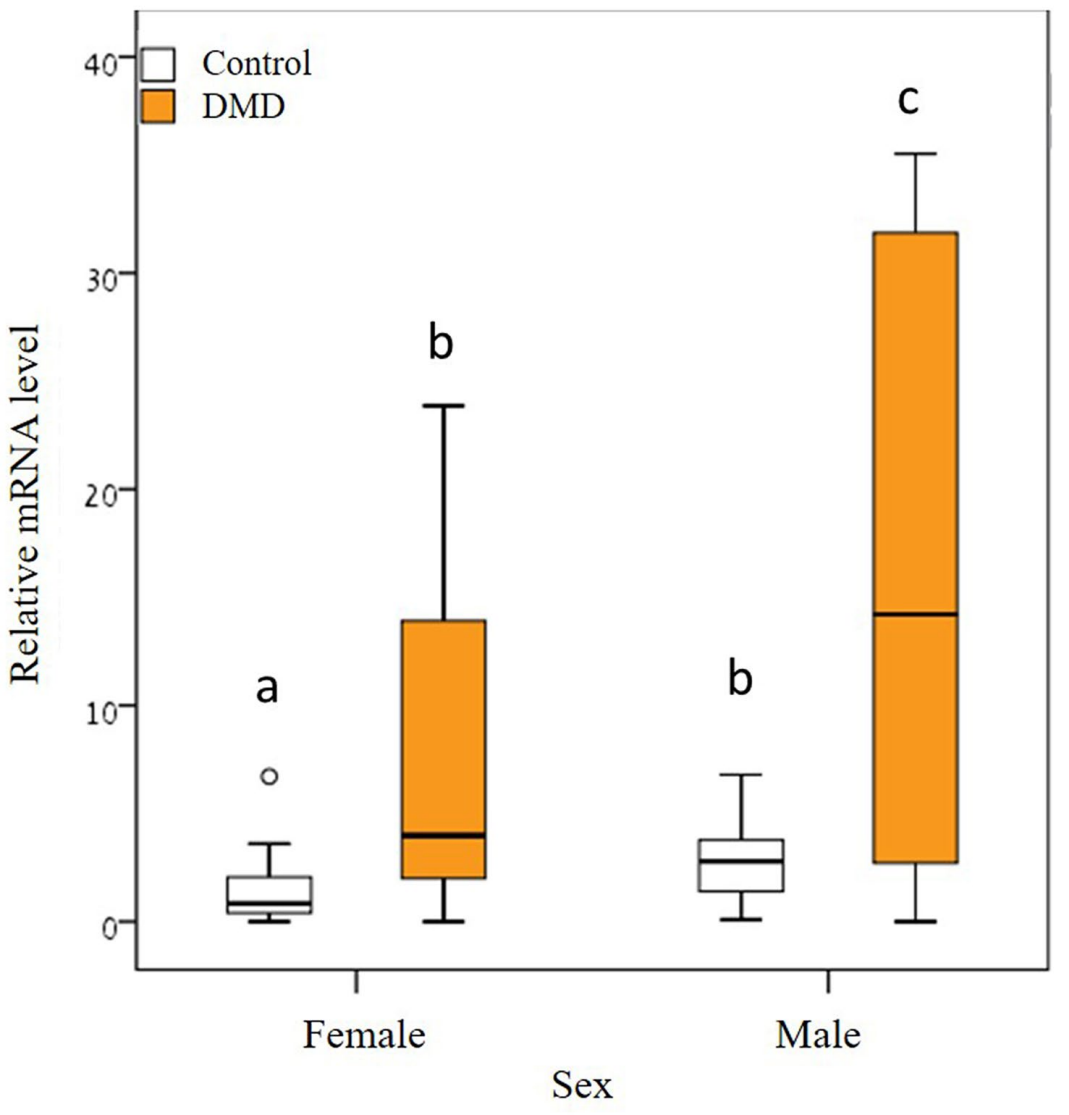
*Tctim* expression in male and female beetles in response to aggregation pheromone; DMD. The synthetic aggregation pheromone increased the expression of *Tctim* (significantly different bars carry different alphabets, Wilcoxon sign rank test, *P* ˂ 0.05). The box encompasses the interquartile range, the line across the box is the median, and the whiskers are drawn to the nearest value within 1.5 times the interquartile range. All remaining outlying points are marked with a circle. Four beetle heads of the same sex were pooled per sample (n). The sample size of the pheromone exposed group was n = 6 and 3 and the control sample size was n = 11 and n = 14 for males and females, respectively.

Exposure of *T. castaneum* to its own pheromone, DMD, led to a significant increase in the expression of *Tctim* (GLM: Z = 5.48, *P* < 0.0001, Fig. 2). This result provides evidence of a feedback regulation at the molecular level of circadian clocks and new insights to possible interactions of social signals on the circadian clock. Both sexes were affected similarly by the exposure to DMD (GLM (treatment*sex interaction): Z = − 0.19, *P* = 0.847, Fig. 2) but male beetles demonstrated significantly higher expression levels of *Tctim* than females (GLM: Z = 2.24, *P* < 0.01, Fig. 2). There was significantly higher variation in gene expression when exposed to DMD (Levene's test of equality F_(1,32)_ = 136.419, *P* < 0.0001). Similar to previous studies, group size positively correlated with gene expression, but this result was marginally non-significant (GLM: Z = 1.94, *P* = 0.052).

#### Behavioral experiment

**Figure 3:**
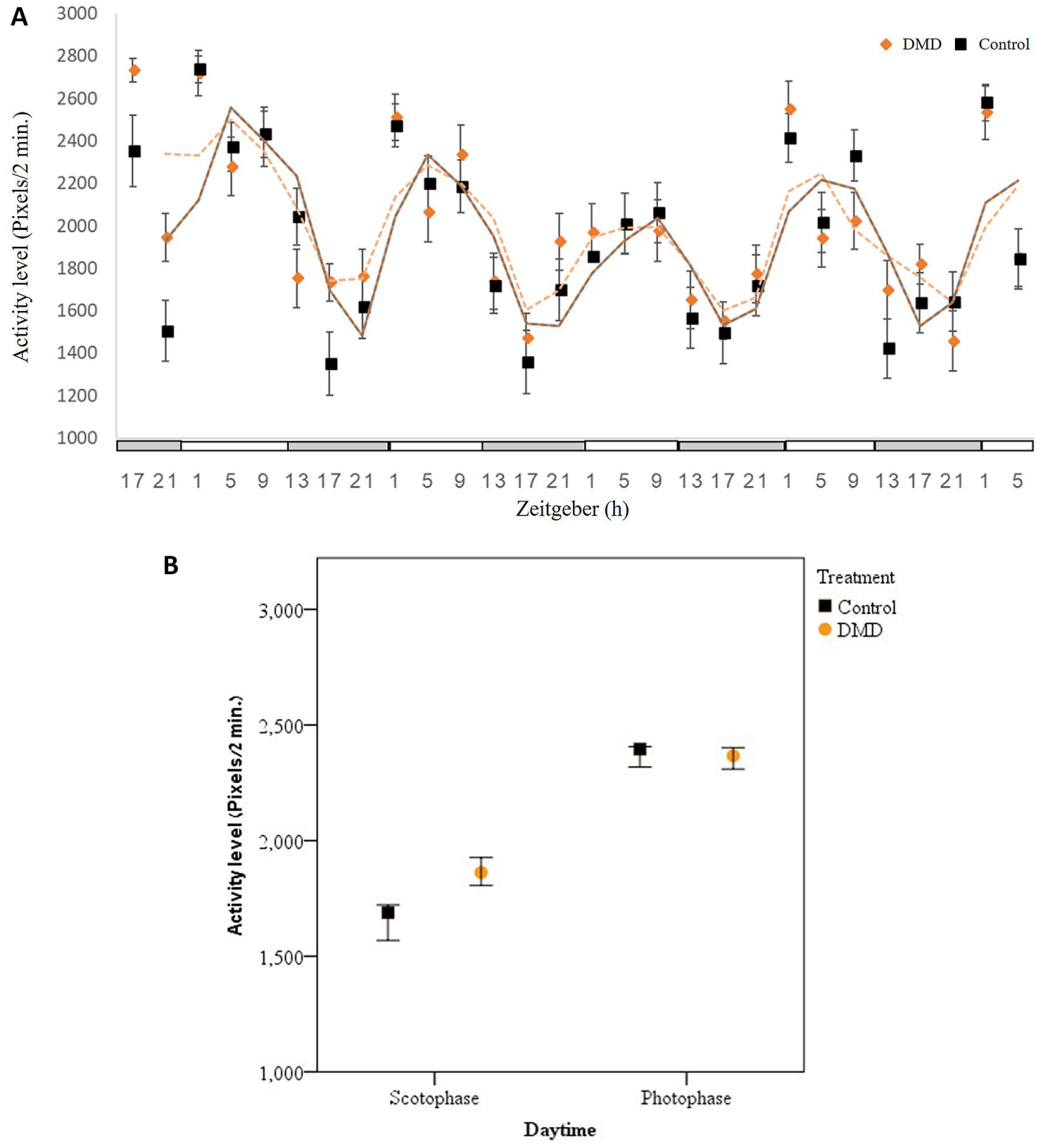
Activity level in response to DMD (mean ± SE). Activity level recording (pixels/2 min.) of control beetles (black trendline) and DMD exposed beetles (red dotted trendline) over five consecutive days (bars below X-axis indicate on lights on (white bar) and lights off (grey bar) phase. In both cases, there is significant rhythmicity with a common peak of activity (Zt 1 to Zt 5, **A**). White and black bars indicate light phase and night, respectively. The synthetic aggregation pheromone increased activity (**B**). This increase is attributed to the dark phase (scotophase).

Both the treatment and the control demonstrated significant rhythmicity with a common peak of activity (DMD: *P* < 0.01, Control: *P* < 0.001) and peak activity time at Zt 1 to Zt 5 (Fig. 3A). Exposure of *T. castaneum* to its own pheromone, DMD, led to a significant higher level of activity when exposed to DMD (χ^2^_(1)_ = 387,669.902, *P* < 0.0001, Fig. 3B). This increase in activity was mainly attributed to the dark phase, i.e. scotophase. At the photophase, the treatment and control did not differ in activity level (Zt 13 to Zt 21, GLM: treatment X time: F(1) = 15.694, *P* < 0.0001, Fig. 3B). In addition, at all time units there was no interaction between the treatments (control and DMD) and the day of activity on the activity level (*P* > 0.726 and *P* > 0.163, respectively).

## Discussion

Our finding that an aggregation pheromone (DMD) has an effect on a circadian clock gene is novel. Previous studies provide evidence that an exposure to pheromones may synchronize the circadian behavioral rhythms^34,35^. In honeybees, the cues that mediate social synchronization are so far unknown^23^ at the molecular level, and the effect of the pheromones was studied on genes other than the circadian genes^36–42^. This study provides direct evidence that an exposure to a species-specific pheromone leads to overexpression of a circadian gene and an increase in the activity level, mainly during the scotophase (dark period). Intriguingly, the variance in *Tctim* expression in the treatment samples is greater than in the control. Although it can be a direct outcome of smaller sample size, it can also indicate the effect of prolonged exposure to non-rhythmic DMD. In the control group, if individuals are exposed to the male's natural DMD, DMD emission is expected to occur at a unique time point (as evident in related species, *Tribolium confusum*^43^). Thus, with no other origins of DMD, individuals are expected, if synchronized by the stimulus, to act approximately at similar times. But when DMD is emitted around the clock (under experimental conditions), the DMD might stimulate individuals at different time points. This in turn should lead to asynchrony and a high variation of gene expression at any sampled point. In the current study, although there is high variation in gene expression, the individuals maintain rhythmicity. This can occur if there is a limited time window, in which individuals are sensitive to DMD stimulus. In this case, prolonged exposure to DMD may stimulate only within the favorable time window inducing an increase in gene expression while maintaining rhythmicity.

*Tctim* overexpression at Zt 13, when exposed to DMD, may either indicate a higher amplitude at the circadian molecular clock or represent overexpression during the whole day. Our results, which show stronger rhythmicity in larger groups, provide strong support for the former option, i.e. a higher amplitude at the circadian molecular clock. In both cases, a change in the circadian clock due to high DMD suggests that the pheromone may create a nested feedback loop^44^. In *T. castaneum*, as in a broad range of species, there is evidence that the pheromone release is governed by the circadian clock^45,46^. We bring evidence that DMD signals back to the circadian clock and alters it. Thus, the circadian clock output can further mediate the circadian clock through various paths^47^. There is accumulating evidence in mammals that feedback from clock-controlled activities may entrain the circadian clock^47^. For example, behavioral arousal may play an important mediating role in the social synchrony of circadian rhythms^47^.

There is a need to apprehend the *T. castaneum's* population spatial dynamics in order to understand the adaptive value of the current feedback system and the crucial role of circadian plasticity. *T. castaneum* has a density-dependent aggregative behavior, expressed by attraction of fewer individuals to higher densities than to lower densities. Once the aggregation exceeds a certain quorum, some individuals disperse and aggregation is therefore confined to a certain density and time window. Within this time, individuals that change their previous behavioral pattern, i.e., searching for the aggregation and releasing DMD for the benefit of mating, will increase their mating success^46^. The plasticity of the circadian clock might be the mechanism underlying these behavioral and physiological alternations.

Although male and female *T. castaneum* beetles have similar responses to DMD, i.e. overexpression of the *Tctim* gene when exposed to DMD, *Tctim* expression is higher in males. This could be attributed to several non-mutually exclusive mechanisms: (1) males may use different expression or binding affinity to the odorant binding proteins or odorant receptors in the antennae^46^. (2) Variations in ovarian hormones may affect clock gene expression in the female brain, introducing additional levels of complexity on the rhythmic modulation of behavior and metabolism^48^. (3) Males and females may differ in their rhythmic pattern^49,50^ while exposed to DMD.

The detected DMD effect on the circadian clock indicates that DMD regulates various biochemical, physiological, and behavioral processes. These processes must be synchronized with the insect’s immediate social environment. For example, in *Tribolium* sp. there is evidence that oxygen consumption^51^, pheromone release and response^20,21^, and flight activity^52^ are the outcomes of the circadian clock. Further studies using functional genomic screening tools (e.g. RNA-interference/ CRISPR) will promote an insight into the functionality and characteristics of the circadian clock. This methodological approach will illuminate the disparity between gene expression pattern demonstrated here and in Li et al^24^. Our results exhibit rhythmic expression of *Tctim* and *Tcper* mRNA levels with a peak at Zt 9 and Zt 13, respectively (Fig. 1B). However, in Li et al^24^ both genes peak at Zt 4. These phase- differences can be accounted as a result of several factors including different strains^53^, different light dark cycle (in Li et al., although collected in 12:12 h L:D regime they were reared and habituated at 14:10 h L:D), light strength^22^ and wavelength^22^, and group size (as evident in the current study). Furthermore, the phase –decoupling between the genes can reflect temporal differences in mRNA dynamics between different body parts^54^ and different environmental cues^55^ (e.g. temperature entrainment).

The findings of this study have an applicative contribution to integrated pest management. *Tribolium castaneum* is currently managed via pheromone-baited traps that attract them. Despite the aforementioned evidence for the importance of pheromone communication to *T. castaneum,* the effectiveness of the traps in laboratory studies and in field documentations are variable^18,56^. We suggest that an artificially induced long exposure to high concentration of DMD may lead to the reduction of the pest fitness by obscuring the circadian clock and in turn will reduce trap catches. In the current study, prolonged exposure to DMD led to higher activity during the dark hours (scotophase). This may exhaust the beetles and contribute to reduced fitness. Thus, changing the well-adapted "alarm clock" to an irregular circadian clock, using DMD is expected to be much more effective in the management of this pest. This is one of the main approaches of the Agro-chronobiology concept of "treating the time", in the integration of chronobiology to agriculture^57^. To conclude, given the importance of circadian clocks for insect reproductive success^50^, the effect of the aggregation pheromone on *Tctim,* a core circadian clock gene, and its fitness consequences should be taken under consideration in future studies of integrated pest management.

## Data Availability

The datasets generated and analyzed are available within the article and from the corresponding author on reasonable request.
